# ‘Our biology is listening’: biomarkers as molecular vestiges of early life and the production of positive childhood experiences in behavioral epigenetics

**DOI:** 10.1057/s41292-024-00337-y

**Published:** 2024-08-31

**Authors:** Robbin Jeffries Hein, Martine Lappé, Fionna Francis Fahey

**Affiliations:** 1 UCLA Institute for Society and Genetics, 621 Charles E. Young Dr., South, Box 957221, 3360 LSB, Los Angeles, CA USA; 2 Social Sciences Department, California Polytechnic State University, San Luis Obispo, 1 Grand Avenue, Building 47, San Luis Obispo, CA USA; 3 Anthropology Department, Purdue University, 700 W. State Street, West Lafayette, IN 47907 USA

**Keywords:** Epigenetics, DOHaD, Biomarkers, Early life adversity, Positive childhood experiences, Epistemic objects

## Abstract

The sciences of environmental epigenetics and the Developmental Origins of Health and Disease have become central in efforts to understand how early life experiences impact health across the life course. This paper draws on interviews with epigenetic scientists and laboratory observations in the United States and Canada to show how scientists conceptualize epigenetic biomarkers as *molecular vestiges of early life* and the consequences this has for postgenomic approaches to health, risk, and intervention. We argue that this process demarcates early life as the optimal time to study and intervene in health and positions biomarkers as conceptual and methodological tools that scientists mobilize to reimagine early life environments. These environments include Positive Childhood Experiences (PCEs), which reflect an emergent and increasingly prominent epistemic object in behavioral epigenetics. Though distinct from widespread research on Early Life Adversity, we show how PCEs continue to essentialize experience in gendered and individualized ways. Further, this paper suggests that focusing on biomarkers as molecular vestiges of early life allows scientists to create stability despite ongoing epistemological and biological unknowns in epigenetics and DOHaD. Our findings contribute new perspectives to social studies of epigenetics, biomarkers, and the production of novel epistemic objects in postgenomic knowledge practices.

## Introduction

*There does indeed seem to be something about the early life environments, whether those are things like socioeconomic status or stress in the family, that seem to get under the skin. I think they provide the best evidence to really think about how our society, how our families work, and how perhaps, really, there is a molecular vestige or molecular imprint of the things we experience in our daily lives, particularly the child’s experience (Participant 23, 02-22-2018).*

The sciences of environmental epigenetics and the Developmental Origins of Health and Disease (DOHaD) have become increasingly central in efforts to understand how environments impact health and illness across the lifecourse (Landecker and Panofsky 2013). Today, researchers from multiple disciplines, including the medical geneticist quoted above, use approaches informed by these fields to study associations between environmental exposures and long-term outcomes, focusing on how early life experiences shape future health. While definitions vary, environmental epigenetics lends itself to these efforts, as it focuses on how “experience gets under the skin” by identifying molecular modifications that affect gene expression without altering DNA sequence (Aristizabal et al. 2019). This reflects a postgenomic orientation toward understanding health and illness as a consequence of complex temporal and multiscalar interactions between genes and environments, which has decentered “genes as central explananda of life and health” (Chiapperino 2024, p. 2; Griffiths and Stotz 2013; Pickersgill 2016; Richardson and Stevens 2015). DOHaD also emphasizes the impacts of environmental experiences and exposures but explicitly locates the foundations of health and disease during fetal development and, to a lesser degree, early childhood (Barker 1990, 1995, 2007; Barker et al. 2010). This shared emphasis on early life is central to our analysis, which focuses on how these sciences shape conceptualizations of health, risk, and intervention.

In this paper, we ask: how are early life experiences and environments being (re)imagined through the sciences of epigenetics and DOHaD, and what consequences does this have for postgenomic approaches to health, risk, and intervention? To answer these questions, we focus on research within the subfield of behavioral epigenetics, which explores how early life experiences impact neurodevelopmental, behavioral, and mental health over the lifecourse (McEwen 2013; McGowan and Szyf 2010; Weaver et al. 2004). Our analysis draws on interviews conducted from 2016 to 2018 with epigenetic scientists studying children’s behavioral health, and our ongoing observations of research practices across laboratories and pediatric health initiatives in the United States and Canada. While the scientists in our study come from numerous disciplines, all shared an initial interest in how experiences of Early Life Adversity (ELA) impact long-term health and health-related behaviors. Experiences of ELA, including chronic poverty, sexual and physical abuse, emotional neglect, and parental substance abuse, have been widely associated with poor physical and mental health outcomes in later life (Hughes et al. 2017; Shonkoff et al. 2022). This area of research is informed by studies in the late 1990s that focused on how “Adverse Childhood Experiences” (ACEs) negatively impact health over the lifecourse (Felitti et al. 1998). Since then, ELA has become a significant focus of children’s health research across pediatric and biomedical fields (Duffy et al. 2018; Garner et al. 2012). Our findings reflect how researchers conceptualize these and other early experiences as meaningful for health, as well as the epistemic objects that emerge as a result.

We argue that scientists’ focus on epigenetic biomarkers—chemical markers that modify gene expression—is central to their models of health, risk, and intervention and notions of what environments matter most during early life (Buklijas 2018). Drawing on our interlocutor’s evocative phrasing in the epigraph, we show how researchers conceptualize these chemical modifications as *molecular vestiges of early life*. This term echoes the notion of “epigenetic vestiges” introduced by Essex et al. (2013), which refers to the potentially enduring changes in gene regulation caused by early life experiences. We build on this phrasing to show how our interlocutors positioned epigenetic biomarkers as important indicators of early life experiences in their models of health, shaped their approach to early life interventions, and the implications this has for the molecularization of environments (Niewöhner 2011). Scientists’ conceptualization of epigenetic biomarkers as molecular vestiges of early life positions them as evidence of the past and potential sites for future intervention. This, we argue, produces a sense of material stability in the context of biological flux and epistemological uncertainty that characterizes postgenomic science and influences approaches to health, risk, and intervention in three important ways.

First, scientists’ conceptualization of biomarkers as molecular vestiges of early life demarcates pregnancy and the first five years as the optimal time to understand the biological effects of experiences and exposures, identify risks, and intervene in future health. Second, and notably, our analysis reflects how this positions epigenetic biomarkers as conceptual and methodological tools that scientists mobilize to expand the early life environments that matter in questions about health. This led the scientists we followed to focus on “Positive Childhood Experiences” (PCEs), which are defined in pediatrics and children’s health literature as “positive events, activities or situations that enhance a child’s life, promoting flourishing, and successful health and developmental outcomes” (Guo et al. 2022, p. 943; Shonkoff et al. 2021). While the effects of ELA are well documented, the benefits of PCEs remain poorly understood (Bethell, et al. 2019; Guo et al. 2022, p. 943; Shonkoff et al. 2021). Since our interviews, PCEs have further become a burgeoning area of research (Han et al. 2023). We show how scientists at our sites mobilize complexities surrounding epigenetic biomarkers to focus their studies on how “safe, stable, and nurturing relationships” may epigenetically “buffer” children from the effects of ELA and promote healthy development (Garner et al. 2021, p. 2; Sege and Browne, 2017, S79). While distinct from ELA research, we argue that through scientists’ focus on biomarkers, studies of PCEs nevertheless continue to emphasize early life and molecular evidence in ways that essentialize experience in highly gendered and individualized ways. Our analysis therefore highlights PCEs as an emergent and increasingly central epistemic object in behavioral epigenetics, drawing attention to a site that has garnered little attention in social studies of health, science, and medicine. Finally, we illustrate how this emphasis on epigenetic biomarkers allows scientists to capitalize on ongoing epistemic uncertainties in epigenetics and DOHaD, leveraging those tensions to produce new research questions and sites of study that nevertheless continue to emphasize problematic notions of health promotion and disease prevention, despite the emphasis on positive experiences. Our findings therefore offer a case study of how scientists conceptualize epigenetic biomarkers as powerful indicators of past exposures and future risk in ways that shape the possibilities for intervention and the novel epistemic objects that emerge in the process.

### Biomarkers and the molecularization of early life experiences

This section provides the conceptual framework for understanding how scientists conceptualize epigenetic biomarkers as molecular vestiges of early life and why this matters for health, risk, and intervention in the postgenomic era. We begin with an overview of epigenetics, DOHaD, and their intersections with ELA research. We then consider STS scholarship related to epigenetic models, biomarkers, and epistemic complexity. Our findings contribute new dimensions to these areas of scholarship by identifying how novel epistemic objects emerge through the sciences of epigenetics and DOHaD (Rheinberger 1997). Historian of science Hans-Jörg Rheinberger characterizes epistemic objects as ‘things’ marked by “their opacity, their surplus, their material transcendence…which is what arouses interest in them and keeps them alive as targets of research” (2005, p. 405). He also contends that these objects “are epistemic by virtue of their preliminarity…because it has not yet been determined whether they will become obsolete as targets of research, or whether they will become transformed into stable, technical objects” (2005, p. 406). By paying attention to epigenetic biomarkers and how scientists conceptualize them, we show how shifting notions of health are being materialized to produce novel epistemic objects in the form of PCEs. These objects of inquiry shift models of early life experience, even as scientists navigate uncertainties associated with them. We argue that PCEs therefore emerge as novel epistemic objects that help generate new research questions while continuing to build significance for the existing ‘experimental systems’ scientists rely on (Rheinberger 1997; Nelson 2012). Our analysis shows how this takes place, and here we discuss the scholarship central to our argument.

Epigenetics is an exemplary and contested field of postgenomic science (Pickersgill et al. 2013). This is in part because epigenetics offers a distinct understanding of health and development that has emerged since the sequencing of the human genome, which sees bodies as mutable, “impressionable” (Meloni 2019), and affected by experience in potentially lasting ways (Chiapperno, 2024; Lappé and Landecker 2015; Meloni 2018; Niewöhner and Lock 2018; Pitts-Taylor 2016). This description reflects a shift from understanding genomes as “static” and unchanging to “reactive” and “exquisitely sensitive” to their environments (see Jablonka 2013; Jablonka and Lamb 2006; Keller 2014, pp. 2423–2427; Lock 2017; Lock and Palsson 2016). As a result, epigenetics has increasingly animated the “scientific and societal imaginary” and shaped understandings of how social and material environments, rather than genes alone, impact health, risk, and health-related interventions (see Darling et al. 2016; Meloni and Testa 2014, p. 436; Lloyd and Müller 2018; Lock 2015).

Questions of how and when an organism is most susceptible to the effects of environmental experiences and exposures and their potentially lasting impact on health and development are also central to DOHaD. A central principle of DOHaD is that embryonic and fetal development represent a particularly susceptible period during which the growing fetus is uniquely vulnerable to environmental exposures (Burggren and Mueller 2015; Penfield and Roberts 1959). Consequently, these very early periods of development are considered foundational for long-term health. Increasingly, scientists have also extended these ‘critical windows’ to include childhood and how experiences of ELA, in particular, can ‘get under the skin’ to affect biological and physiological processes during these early periods (Aristizabal et al. 2019; Burggren and Mueller 2015). For example, results from epigenetic studies show how experiences of ELA can negatively impact brain development, the nervous system, and biological pathways of ‘normal’ development (Barker et al. 2010; Chung et al. 2016; Gluckman and Hanson 2006). In turn, this may lead to deleterious and long-lasting impacts on health and health-related behaviors (Champagne 2010; Gudsnuk and Champagne 2012; Kundakovic and Champagne 2015; Meaney et al. 1994; Meaney and Szyf 2005; Shonkoff et al. 2009; Weaver et al. 2006). These include an increased risk for anxiety, depression, and other outcomes that are often characterized as the result of “developmental programming” (Barker 1995, 1998) or “developmental conditioning” during sensitive windows of development (Hanson and Gluckman 2014). Consequently, concerns about the health impacts of ELA are of growing interest in epigenetics and DOHaD research and are central to the sites we discuss in this paper (Forkey et al. 2021; Garner et al. 2012, 2021; Hughes et al. 2017; Mulligan 2016; Shonkoff et al. 2009, 2022).

The rise of epigenetic and DOHaD studies, and growing questions about the scientific and cultural ‘hype’ associated with them have also become topics of increased scrutiny for many STS scholars (Meloni and Testa 2014). Specifically, scholars have examined how molecular accounts of the social often embody essentialized and reductionist representations of lived experience (Chiapperino, 2018; Lock 2015; Niewöhner 2011, 2020; Niewöhner and Lock 2018; Romjin and Louvel 2021; Penkler 2022). For example, scientists often rely on biological proxies for social experiences and use crude survey instruments to demonstrate the biological embedding of multifaceted experiences (Lappé et al. 2022; Lock 2015; Mansfield 2012; Pitts-Taylor 2016; Roberts 2021; Valdez 2019). This often reduces the complexity of social and material environments and their entanglements into discrete variables that are removed from their social and political contexts (Kenney and Müller 2016; Lock and Palsson 2016).

Feminist scholars of science in particular illustrate these tendencies, arguing that epigenetic and DOHaD research often reproduces individualized narratives of ‘good’ motherhood and emphasizes maternal responsibility for future health in ways that reinscribe sexist and racist tropes (Lappé, 2016; Richardson 2015, 2021; Valdez 2018, 2021; Warin et al. 2011). This is evident in rodent and human studies that focus on the epigenetic impacts of maternal behavior and early caregiving on offspring health and in research on the intergenerational effects of stress, trauma, and adversity (Kenney and Müller 2016; Lappé et al. 2022; Lappé and Jeffries Hein 2021). These findings reflect how epigenetic and DOHaD studies often diminish the impact of broader systems and social environments that influence health and illness (Lappé, 2018; Valdez and Lappé, 2024). Further, scholars show that even when concerns about racism and inequity are central to research on the intergenerational transmission of trauma, dominant epigenetic methods and modes of evidence often diminish the importance of historical and structural oppression (Davis 2019; Kuzawa and Sweet 2008; Roberts and Rollins 2020; Saldaña Tejeda and Wade, 2019). These findings connect to broader concerns about “epigenetic determinism” (Waggoner and Uller 2015) and “somatic determinism” (Lock 2013) that have emerged throughout social studies of the postgenomic sciences, despite its promise to embrace complexity (Nelson 2018; Penkler 2022; Rheinberger 1997). In these ways and others, the sciences of epigenetics and DOHaD have become critical sites that are actively shaping how early environments matter in conceptualizations and practices related to health, risk, and intervention. (Lappé et al. 2019; Lloyd et al. 2022; Pentecost et al. 2018).

Importantly for this paper, social science scholars have also explored the science and implications of biomarkers as tools for generating diagnoses, prognoses, and predictions across different disease areas, including the challenges they pose for therapeutic and clinical translation (see Harris and Schorpp 2018; Pinel et al. 2017). These include the role of biomarkers in public health concerns around the global aging “pandemic” and the etiology and prognosis of neurodegenerative disorders (Lock 2007, 2013; Müller and Samaras 2018; Milne and Latimer 2020) as well as demonstrating links between poor nutritional environments and the ‘obesity crisis’ (Benyshek 2013; Guthman and Mansfield 2012; Landecker 2011, 2016; Warin et al. 2016). In addition to illustrating how environments become biologically embedded, scholars have also highlighted the role of biomarkers as “technologies of knowing” (Arteaga Pérez 2021, p.5), “devices for naming and identifying’’ biosocial processes (Crabu 2016, p. 315), their effects on managing disease risk (Filipe et al. 2021; Lloyd et al. 2022; Meloni 2014), and in shaping patient subjectivities (Adams et al. 2009; Sulik 2009). These findings provide an important foundation for our analysis, as they signal the power that biomarkers have in shaping understandings of health, risk, and intervention across the health sciences. As numerous STS studies of epigenetic biomarkers, early life experiences, and psychiatric risk show, and our argument here reflects, there nevertheless remains a “limited understanding of causal relationships between epigenetic molecular modifications, complex emotional and affective states, and multiscalar biological properties mediating their interplay” (Lloyd et al. 2023, p. 9; Lock 2007, 2013; Nelson et al. 2018).

STS scholars have shown how such epistemic uncertainties, “ontological ambiguities,” and the instability of knowing associated with epigenetics, as well as its appreciation of bodies as malleable, have become accelerators for new research questions and empirical sites of study (Pickersgill 2016, p. 191; Lloyd and Raikel, 2018; Rheinberger 1997). As sociologist Martin Pickersgill (2016) argues, “conceptual lability and instability do not necessarily impede biomedical innovation, but can instead drive it forward” (p. 198). Sahra Gibbon (2018) illustrates this in her work on cancer and risk. She highlights the “gaps, spaces and uncertainties’’ that help to generate novel epigenetic environments amidst what she describes as an “inchoate and unfolding terrain of understanding” in cancer research (p. 761). In his recent analysis of biomarkers in the epigenetics of stress, Luca Chiapperino (2024) also argues that a process of “complexification” occurs as scientists navigate “ontologically productive tension[s]” that enable them to reconfigure experimental systems to “better” understand biosocial processes associated with health (pp. 4–5). Similarly, as we describe below, the scientists we follow harness the complexities and tensions in epigenetics and DOHaD logics to generate novel research questions and sites of inquiry and, in doing so, build greater significance in their existing experimental systems (Niewöhner 2011; Nelson 2012; Pickersgill 2020). This reflects how their “experimental arrangement” is, as Hans-Jörg Rheinberger (1997) writes, “sufficiently open” to absorb and capitalize on complexities that arise through their research. Producing and validating the importance of epigenetic biomarkers therefore allows scientists to remain firmly situated in their conceptual and methodological frameworks despite ongoing unknowns (Latimer and Hillman, 2019; pp. 80–81; Niewöhner 2011; Nelson 2012).

We argue that through the continued conceptualization of biomarkers as molecular vestiges of early life, scientists leverage epistemic uncertainties in epigenetics and DOHaD to produce an emergent epistemic object in PCEs. While this is all done in the name of ‘better’ demonstrating how early life experiences impact health, these practices also produce material stability in the context of changing biological and epistemological conditions. What results is a continued emphasis on molecular knowledge and an individualized and gendered sense of what environments matter for health. Our analysis below traces these processes and the persistent promise of biomarkers that remains at their core.

### Methods

Our findings draw on a multi-year, multi-sited ethnographic study (Marcus 1995) focused on the production and translation of epigenetic knowledge related to children’s neurodevelopmental, behavioral, and psychiatric health across two university laboratories in the US and Canada and numerous observations at conferences and meetings related to child health, development, and epigenetics between 2016 and 2024. Our laboratory observations included participant observation of research activities and experiments, in-person and virtual attendance at lab meetings, informal discussions with lab members, and correspondence with scientists about publications, grants, and public health or community initiatives they were involved in. We complemented these observations with attendance at scientific conferences related to epigenetics and children’s health and ongoing participation in efforts by scientists, community representatives, and pediatricians to develop clinical tools to document and address the health effects of early life adversity. The scientists we observed and interviewed included postdoctoral researchers, mid-career principal investigators, full professors, lab staff, and technicians, and their disciplinary training spanned numerous fields, including developmental psychology, psychiatry, epidemiology, biochemistry, molecular biology, genetics, biochemistry, and pediatrics.

Using grounded theory methods (Charmaz 2006; Glaser and Strauss 1967; Strauss and Corbin 1994), the sections below reflect our analysis of 40 in-depth interviews with epigenetic scientists who were part of this broader study. These results are also informed by our observations as noted above. The formal interviews focused on scientists’ training, research, study designs, views and definitions of epigenetics, interactions with media, and their work’s social and ethical implications. While scientists’ training and research designs varied, all emphasized the potential for epigenetics to advance understandings of the relationships between early life experiences, the molecular underpinnings of children’s neurodevelopmental, behavioral, and mental health, and long-term outcomes. In the sections below, we explore how biomarkers figured centrally in these efforts, the emergence of PCEs in epigenetic research, and how scientists mobilize ongoing uncertainties in postgenomics to generate new research inquiries. This section is followed by our discussion of how these findings relate to existing literature in STS and their impacts on conceptualizations of health, risk, and intervention.

### Identifying epigenetic biomarkers: “that’s the dream”

Scientists we interviewed approached their studies with an emphasis on how environments and experiences in utero and during early life influence health and development over the life course. As one researcher aptly put it, “Our biology is listening. Our body is very much in tune with our environment” (Participant 17, 08–23-2017). Many described epigenetic biomarkers as the “best evidence” of how early experiences and environments become embodied and for understanding their potential impacts on future health (Participant 23, 02–22-2018). As a result, scientists focused on early life as a critical period for documenting the biological impacts of experience and as a particularly effective window for intervention. Through this logic, biomarkers were positioned as molecular vestiges of early life, thus holding the potential to indicate past exposure, predict risk, and shape both individual and population-based interventions, if only they could be identified.

A developmental psychologist studying the effects of extreme early life stressors, including physical abuse and neglect, on a propensity toward aggression in adolescence described how biomarkers could inform potential “care and intervention” strategies. She explained

I think biomarker potential is something that a lot of people are interested in. Being able to potentially find biomarkers for risk, for psychiatric risk, [that] might inform strategies for care and intervention. I mean, these are things that in the future people are hoping, right, that will help personalize strategies to support people or reduce their disease risk later in life*.* (Participant 38, no date)

Building on the logic of biomarkers as molecular vestiges of early life and therefore the connection between lived experiences and outcomes needed to indicate future health, the medical geneticist and epigenetics researcher quoted in the epigraph described their translational potential as follows:

In terms of application, we can perhaps develop a better and different diagnostic test to spot kids that might be at risk for developing disorders early on [and] then, hopefully, provide interventions that tend to work well if they’re started early with the help of these epigenetic measures. (Participant 23, 02-22-2018)

His account reflects how the identification of biomarkers became central to new prevention efforts, even before such diagnostic tests were developed.

Another developmental psychologist analyzing the effects of maternal stressors associated with preterm birth similarly described epigenetic modifications as “the best markers for environmental exposures and developmental trajectories” (Participant 29, 09–05-2018). She explained the anticipated importance of biomarkers in shaping interventions for infants and youth, stating:

If epigenetic markers really tell us something about how adverse conditions affect our developmental trajectories, I will be able to provide interventions that are counterbalancing to the adverse epigenetic changes or other kinds of epigenetic factors. (Participant 29, 09-05-2018)

Like the scientists above, her statement positions epigenetic biomarkers as holding significant potential to shape knowledge about the developmental impacts of adversity and future interventions to “counterbalance” them.

An environmental epidemiologist also embraced the idea that biomarkers could help identify at-risk children by providing a basis for new screening technologies. She explained this possibility in light of her work as a maternal–fetal clinician-researcher studying the intrauterine environments of pregnant women who experienced trauma associated with September 11, 2001. When describing the study, she said the “purpose was to look at exposures associated with being in utero near the World Trade Center as it was burning” (Participant 18, 08–29-2017). To carry out these studies, her research team designed several prospective cohort and sibling studies that followed mothers and their children from pregnancy through late adolescence to identify epigenetic modifications associated with this specific type of *in-utero* stress. Using biological samples from expectant mothers and their children following birth, she explained how methylation patterns could help shape future population-based screening programs:

The thing we have been playing around with more recently is to try to see whether we can use methylation patterns as a biomarker. So more than trying to say, ‘Is this mark on the mechanistic pathway?’—I don’t particularly care what gene it’s on–if it predicts exposure really well, then maybe we don’t have to measure exposure in all kinds of kids. We can just look at their methylation patterns and tell you with some degree of certainty whether or not they’ve been exposed or whether or not they’re at risk for some outcome down the line. We’re just not there yet, but that's the way we’re thinking about it, whether it can be used as some kind of screening tool, but it’s work, [and] we’re pretty far from that, honestly. (Participant 18, 08-29-2017)

This researcher was keenly aware of the difficulties associated with developing population-based screening tools but hoped that biomarkers could figure centrally in the promise of doing so effectively and efficiently.

She went on to describe how establishing and validating a biomarker that reflects epigenetic alterations to telomeres (DNA protein structures that protect the genome) could serve as a “good proxy for an objective measure of adversity” because telomeres are “sensitive to oxidative stress.” As a result, she saw changes to telomere length as providing a molecular indication of “psychosocial adversity.” She explained the efficiency of this approach, stating:

If you can measure methylation, and then I can apply my signature and see who is at risk and then validate that, then that’s an amazing resource…If I could tell you…who’s at risk…then we can target intervention. So that becomes an efficiency that way. Again, we’re not there yet, but *that’s the dream*. If you can figure out who is most at risk, you can develop an intervention. (Participant 18, 08-29-2017, emphasis added)

From the perspectives of our interlocutors, identifying stable and reliable epigenetic biomarkers associated with early life experiences held the potential to shape both the development of population-based screening tools and the direction of individualized interventions. While still a “dream,” the accounts in this section reflect how researchers positioned the discovery and validation of epigenetic biomarkers as central to their science and as a way to identify exactly how molecular vestiges of early life shape the future. Though biomarkers remained elusive and imperfect, scientists nevertheless saw them as the “best evidence” of how early life experiences and environments matter for long-term health, and—as we discuss in the next section—key sites through which scientists also imagined interventions.

### Positive childhood experiences: an emergent epistemic object

While nearly all of our interlocutors focused on the impacts of ELA on children’s behavioral health and well-being, some scientists also focused on the biological impacts of PCEs (Guo et al. 2022). Here, we illustrate scientists’ focus on PCEs as an emergent epistemic object within epigenetics and explore the consequences this has for ideas about health and intervention. This section reveals how epigenetic research on PCEs remains rooted in the promise that molecular biomarkers can provide actionable indicators of risk. However, instead of seeing biomarkers primarily as evidence of past experiences, we show how PCE research envisions them as sites through which biological change also becomes socially actionable and biologically possible.

Reflecting on new research directions in behavioral epigenetics, a molecular epidemiologist explained the importance of studying PCEs for reimagining how health might be investigated. He suggested:

I do think there’s gonna be a little bit of a change to look at more of the positive side of things…So what is good health? How do we define that? So, I think there may be more work going toward that side of it. What are the positive things that we can find so that at least we know where to go? And what do we have to change to get [people] on a good trajectory? That could be where we will see some things. (Participant 7, 04-04-2017)

He elaborated on this potential by noting that in the National Institutes of Health’s (NIH) Environmental Influences on Children’s Outcomes (ECHO) Program launched in 2016, one of its five core areas of research includes “positive health” (NIH, 2023). He described the importance of this from the perspective of his epigenetic research into fetal environments, placenta epigenetics, and growth and behavioral outcomes in children, and wanting to understand how people can be better supported to live healthy lives.

A developmental psychologist who studies maternal caregiving behaviors with infants also emphasized the importance of studying positive environments. She discussed the importance of studying the power of positive parenting styles for children’s health, particularly within contexts of maternal adversity. By drawing a comparison with prominent epigenetic studies of maternal stress and offspring anxiety (See Fish et al. 2004; Meaney et al. 2001; Weaver et al. 2004, 2006), she argued:

We focus so much on this deficit model, [but].clearly, there are plenty of moms with depression, plenty of moms undergoing a lot of stress that are excellent, exceptional parents. We just don’t focus on that group at all. We need to do a better job in the field of looking at *individual differences* in these parenting styles… I think that it’s important to focus on the power that the caregiver has in buffering the child against stress. (Participant 5, 03-10-2017, emphasis added)

She continued to explain her interest and findings, noting the positive buffering effect of maternal sensitivity:

I was interested in maternal depression and [the impact of] maternal sensitivity on epigenetic patterns [in infants]. I found that mothers who had depressive symptoms but were sensitive, had infants with methylation profiles that were similar to infants whose mothers weren’t depressed at all. I think that also points to the power that…positive maternal behavior can have on these infant outcomes. (Participant 5, 03-10-2017)

In this example and others in our study, maternal behavior and sensitivity to children become formalized as a positive environment that affords biological protection to offspring who have experienced adversity. However, positioning such behavior as “powerful” continues to rely on epigenetic evidence of its effects and a sense that such evidence is observable through molecular vestiges of experience.

A behavioral neurobiologist specializing in rodent models of ELA expressed hope that her experimental work illustrating that the “epigenome is pretty malleable” would help inform human studies she collaborated on that used positive parenting interventions (Participant 17, 08–23-2017). She explained the connection between epigenetic plasticity and parenting interventions in the following way: “We can think about parenting intervention [as a way] to change DNA methylation” in children who had been previously maltreated and “then have some positive behavioral outcomes for these kids” (Participant 17, 08–23-2017).

She elaborated on the research collaboration, describing how this type of behavioral intervention involved working with parents to be “more in tune with their children,” including:

Things like follow[ing] their lead and [exhibiting] less frightening behavior. It’s a ten-week program, and with this ten-week program, the outcomes, you see a lot of positive behavioral change in the children. Better emotion regulation, better attachment scores, you see a reduction in some of their cortisol levels where you actually see those children get the nice diurnal fluctuation of cortisol that a normal child will show you. (Participant 17, 08-23-17).

This study, like the maternal-infant interaction research noted above, also utilized epigenetic evidence to reinforce the biological impacts that positive parenting can have, particularly in adverse circumstances. Doing so reinforced the idea that experiences can be embodied in ways that have lasting impacts on health while isolating what experiences were counted as ‘positive’ in the process.

In addition to human studies analyzing the effects of positive caregiving, some researchers also focused on enhancing rodent living environments in ways that facilitated play, nurturance, and safety. These ‘enriched’ environments suggest additional ways that scientists envisioned environments as having the power to affect the epigenome positively. A neuroscientist who studies the effects of housing environments and diet on the stress reactivity of newborn mice highlighted the importance of these material conditions for understanding the foundations of health. He explained:

With mice [dams], we have this semi-naturalistic housing so they can make burrows and everything like that, so it’s a bit more explorative. And then, we have other [mouse] mothers who are raising their offspring in standard housing. And one of the biggest effects, beyond maternal behavior, is through housing. [It is] not just the mother, then, but the environment in which she's rearing her offspring that matters. (Participant 24, 04-05-2018)

This experimental design moves out from the focus on mothers to actively consider and test how the physical environment impacts health and development. In this example, research findings demonstrate the positive impacts of living in an “enriched environment” on pup behaviors (Champagne 2010, p. 28; Gudsnuk and Champagne 2012). Though this design remains highly gendered through its continued interest in maternal behavior, it is important to note how living conditions are also conceptualized as meaningful early environments in this model. This example also provides an important connection between animal studies and our interlocutors’ hopes for human lives, as this researcher explained his work in the context of a society where “poverty, and a lack of resources, or lack of health care, or substandard housing conditions” affects human health (Participant 24, 04–05-2018).

Although PCEs represent novel epistemic objects in epigenetic studies of early life, this section suggests how they remain rooted in epigenetic logics that frame positive environments in limited terms, as we expand in the discussion section below. As a result, both ELA and PCE research reinforce early life as pivotal for future health while continuing to position biomarkers as molecular vestiges that help explain what kind of environments matter and when.

### Epistemic complexity: a cautionary and productive tale

The sections above reflect how biomarkers allow experiences to become molecular vestiges that tell a story about how society works within us. Yet researchers’ ongoing efforts to identify these molecular connections between experiences and outcomes often resulted in challenges. As one scientist quipped, “Epigenetics is a really exciting area, [but] it raises more questions than it answers” (Participant 38, no date). These questions included uncertainties surrounding epigenetic stability, plasticity, and whether documented changes reflected adaptive responses and normal variation or were truly indicative of risk. Below, we explore some of the challenges scientists faced in interpreting their findings in the context of epigenetic and DOHaD complexities and how they mobilized these in the further direction of new studies.

Scientists routinely described epigenetic modifications as plastic and the effects of prior exposures as potentially reversible. This sense of biological possibility was foundational to PCE research in particular and its promise to create meaningful biological change for the better. A neuroepigeneticist specializing in physiological stress responses, neurological functionality, and long-term learning behaviors explained epigenetic modifications as follows:

I think that’s really the whole point of, certainly, histone modifications and, to some degree, DNA methylation, is that these things are actually much more plastic than we used to think… For a long time, the field would always discover a new chemical modification and say, ‘Well, it changes, and it’s there for life.’ I think it’s pretty clear that all these things are very dynamic. I believe that if you can push your system, you know, rewire your system toward one trajectory, then you can probably reverse it as well. (Participant 14, 08-08-2017)

According to this neuroepigeneticist, “things like chronic stress can lead to these [epigenetic] changes that may promote later life susceptibility to depressive-like phenotypes.” However, because epigenetic modifications are “much more plastic,” the effects of harmful exposures are not necessarily set “for life” (Participant 14, 08–08-2017). His example suggests that epigenetic modifications are not so plastic as to never present concern but may also be malleable enough to change. This productive tension between plasticity and programming continues to animate and give relevance to epigenetic and DOHaD sciences. It also drives the push within them to identify valid biomarkers as evidence of when and how this works precisely so appropriate and timely interventions can be developed.

An environmental epidemiologist with a molecular and cellular biology background also characterized epigenetic modifications as dynamic, temporally specific, and differently responsive to exposures. These characteristics positioned epigenetic modifications as moving targets that were nevertheless relevant for assessing risk and disease susceptibility insofar as they were stable enough to be reliably measured while remaining open to change. He explained this tension as follows:

Epigenetic marks are very plastic, meaning that they’re going to fluctuate and change over your life. They’re going to fluctuate and change for any given exposure. It’s just very hard to say, ‘This is your epigenetics, and this is what it's going to be.’ Epigenetics is just one of those things that help regulate all these integral things about how we function. It’s like fingerprinting and looking at certain epigenetic marks that may be related to disease. The idea is that they’re so plastic. They change on a minute, hourly, daily, or monthly basis. That’s how it’s different from genetics. And then, honestly, this idea of reversibility also makes it very distinct. (Participant 6, 03-10-2017)

The dual qualities of epigenetic stability and reversibility provided an enduring pull for many of our interlocutors. While discussing the promises of epigenetics, an epigeneticist trained in psychopharmacology described the impact of epigenetic stability on the discovery of biomarkers and the development of interventions, including those noted in the sections above. She explained

A lot of researchers are really interested in finding some sort of biomarker so that they can implement some sort of intervention sooner. But what I would also really like to find out is how stable these epigenetic markers are. If one forms an epigenetic characterization of an individual, how stable is that over time? Everybody has their own genome, and that’s very stable unless, of course, [there are] mutations. On the other hand, [there is] gene expression, which can really change in response to the environment. Exactly how stable it is and how it could be used, let’s say, as a marker or disease marker—that, I think, still has to really be defined. (Participant 25, 07-13-2018)

Even as she remarked on the uncertainties surrounding epigenetic stability, her research remained focused on establishing an epigenetic biomarker that could be linked to outcomes that were “stable, or at least long-lasting, behavioral abnormalities” (Participant 25, 07–13-2018).

Researchers’ interest in connecting epigenetic changes to later health outcomes was also affected by the dynamic nature they ascribed to epigenetics. According to the developmental psychologist introduced above, epigenetic instability was unsurprising given that epigenetic change reflects a “dynamic process” (Participant 38, no date). She noted:

Things that happen around this very, very early development could have ramifications, or downstream effects, on systems, on biological systems…We know that epigenetics, well, it’s a dynamic process…so it’s not so surprising that they’re not very stable. But how they might affect outcomes at a given time point versus another is something that we really need to look into. (Participant 38, no date)

She expanded on this reflection and highlighted a key concern about how epigenetic adaptations might shape long-term risk. She explained that, through her research:

It was very clear to me, if it hadn’t been already, that there was such a strong connection between very severe stressors and poor mental health outcomes in young people. I became interested in mechanisms, so really thinking about how do these stressors become, for lack of a better word, biologically embedded, or how do they affect the children’s biology in a way that might be adapted in the short term, but really, in the long-term, might increase this risk for mental health outcomes. What this knowledge contributes to is that it informs existing models of things that we already know about—gene-environment interactions or biological embedding, or this idea of latent vulnerability—that experiencing a lot of [adversity] might affect your health for decades, even long after the exposure has stopped. (Participant 38, no date)

Her observations about the potentially enduring effects of adverse experiences reflect how epigenetics can meaningfully shift understandings and approaches to risk and intervention.

According to a molecular epidemiologist who studies the epigenetics of childhood behavioral disorders, epigenetics can also ‘cloud’ understandings of disease risk. Drawing on an example from his earlier work on genetics and mutagenesis in cancer research, he explained

Before, we had classical carcinogenesis models or mutagenesis assays. The chemical was mutagenic. You defined its risk that way. But with epigenetic research, it makes stuff a lot more cloudy because a lot of things might have an epigenetic effect, or some type of effect that may be something that’s going to lead to health risk, but maybe not. It may have more of an adaptive response side of it. (Participant 7, 04-04-2017)

He elaborated on this description, adding that an adaptive response potentially “allows the child to continue to develop relatively normally.” He explained that while it is promising to connect “early phenotypes… to behavioral outcomes in children,” epigenetic modifications may simply reflect “normal variation.” He elaborated on this point, saying:

We’re not focused necessarily on extreme outcomes…Instead, what we’re thinking about is that there’s a normal variation in the population for all of these different behaviors for growth, [and] for most outcomes. So, how can we try to understand what that's about? That’s where I think some of the epigenetics work comes in, and it’s really interesting. It may be able to explain that normal variation. So it’s not so much like an extreme genotype-factor, mutation-type effect that you immediately get an obvious phenotype…[Epigenetic] modifications might be an adaptation response, and some of it may not necessarily be negative. So, I think there’s a lot of negative perception, and part of this is because a lot of epigenetics came from cancer work where everything is bad. (Participant 7, 04-04-2017)

This researcher points out that, rather than inherently problematic, adaptive responses can be appropriate, necessary, or even positive reactions to external stimuli and therefore central to healthy development and survival (Masten 2001; McEwen 1998; McEwen et al. 2013). What he and others are interested in, then, is the degree to which such epigenetic changes reflect “normal” variation and when deviation from this norm becomes problematic. This designation matters deeply for the interventions described in the sections above and the promises of epigenetics as a field.

## Discussion

The findings above contribute new perspectives to social studies of postgenomics by illustrating three ways that epigenetics and DOHaD shape contemporary conceptualizations of health, risk, and intervention. First, we show how scientists conceptualize epigenetic biomarkers as molecular vestiges of early life. This positions epigenetic evidence as the best indicator of biological embedding while also fortifying the idea of early life as a uniquely important period for lasting biological change. We argue that this materializes connections between early life experiences and health outcomes in ways that valorize epigenetic knowledge as a powerful “arbiter of truth” (Lappé et al. 2022). As such, epigenetic biomarkers are reinforced as central in documenting when, how, and which environments matter for health.

As numerous STS scholars have shown, however, the promise that epigenetics would better account for biosocial complexity and its relationships to health has been met with critiques of reductionism, determinism, and essentialism (Penkler 2022). We similarly find that the conceptualization of biomarkers as molecular vestiges of early life produces an ‘ontological flattening’ of complex social experiences and environments by transforming them into chemical signals and discrete variables (Landecker 2016). As we and others have argued, this leads to narrow representations of complex experiences and environments and individualized modes of responsibility that do not account for or alter structural determinants of health (Yates-Doerr, 2020). Despite this, sociologist Ingrid Metzler (2010) argues that, across the biomedical sciences, “we are currently witnessing a ‘biomarkerization’ of health and disease defined as an ongoing future-oriented process that seeks to solve biomedical as well as public health problems through investments into biomarker research at the present time” (p. 407). This is certainly evident in the sciences we study, where scientists deploy biomarkers as central conceptual and methodological tools to reimagine environments associated with health. In doing so, they reinforce the value of molecular knowledge in the name of producing “better” understandings of the etiology and trajectory of health and disease (Chiapperino 2024). In this sense, we have shown how biomarkers are active in changing models of what counts as health and illness and implicit in the emergent interventions that might follow.

Our second finding reflects how scientists’ focus on biomarkers also does more than just molecularize environments and experiences—it allows them to advance new notions of what early environments matter for health through studying PCEs. While many of our interlocutors continued to address the effects of ELA, we show how research on PCEs shifted their science in new directions and oriented studies toward ideas of health promotion as a form of early intervention. This reflects a concerted effort by scientists to identify ‘healthy’ environments and experiences during early life that might contribute positively to development. As scholars in psychology, child development, and other related fields have argued, this approach runs counter to ELA models, which are “problem-based” and focus on “what happens biologically in the absence of mitigating social and emotional buffers” (Garner et al. 2021, pp. 1–2). In contrast, these scientists and those we study see PCEs as a “solution-focused” approach focusing on safety, stability, and the importance of nurturing relationships, which may also mitigate the biological effects of early adversity (Garner et al. 2021, p. 2). This shift is intended to provide a corrective to deficit models of health in ways that “could reprioritize clinical activities, rewrite research agendas, and realign our collective advocacy” related to health (Garner et al. 2021, p. 1). In these ways, PCEs reimagine notions of health, risk, and intervention “beyond the current epistemic horizon by bringing the social and material environment into molecular research” in novel ways (Niewöhner 2011, p. 289). We have argued that PCEs therefore reflect an emergent epistemic object in environmental epigenetics that deserves the ongoing attention of STS scholars.

However, our analysis above delineates the limits of these objects in important ways. We have argued that despite their imagined potential, PCEs continue to build on established experimental systems associated with ELA in ways that produce limited notions of health, risk, and intervention. This includes concerns about the reductionism of complex experiences and early life experiences and the reification of maternal responsibilities for child health. Further, in the PCE models we discuss, other important and proximate influences on health, including paternal effects, are overlooked (Almeling 2020; Chiapperino and Panese 2018; Pentecost et al. 2018; Sharp et al. 2018). Significantly, as with models of ELA, the broader social circumstances that affect individual and family lives are also obscured through the PCEs our interlocutors study (Lappé and Jeffries Hein 2021; Pentecost et al. 2018; Richardson 2015, 2021; Warin et al. 2016). This can be seen in the models emphasizing maternal care as primary in shaping child health. Further, even when living conditions were central to researchers’ work in rodent studies of enriched environments, we have shown how conceptualizations of positive environments remained confined to measurable factors, including bedding, light, food sources, and opportunities for play. These limitations suggest that PCEs remain rooted in epistemic and molecular logics that reinforce STS scholars’ call for caution regarding epigenetics’ “promissory notes” (Rapp 2018, p. 785).

Finally, our analysis reflects how scientists also mobilized epigenetic biomarkers to navigate epistemic complexities in ways that moved their fields forward and expanded their research (Nelson 2012). As our interlocutors suggest, epigenetics produces as many questions as it answers. Faced with an “epistemology of the imprecise” (Rheinberger 2000), we show how scientists responded to uncertainties around plasticity, adaptation, and variation by generating novel interpretations of early life experiences and their impacts on health, including the possibility of positive adaptation and variation associated with adverse exposures. These considerations shaped scientists’ understandings of the etiology and course of health and illness, including the role of resilience in epigenetic models of ELA and as a rationale for PCE studies. We argue that these approaches reinforce biomarkers as central to documenting biological change. Therefore, even as the possibility of adaptation and normal variation pose challenges to the promises of epigenetics, we show how scientists transform these epistemic uncertainties into opportunities by identifying new research questions and explanatory logics, and by proposing new interventions associated with them. As we have shown, this occurs through conceptualizing and expanding biomarkers into new research areas. Our analysis therefore reflects how scientists depend on biomarkers as evidence of the connections between data on early experiences and their relevance for health outcomes, even in the face of uncertainties. As a result, biomarkers provide epistemic stability among shifting landscapes of epigenetic ambiguity and biological flux. Their production and validation therefore remains an elusive yet highly sought-after endeavor in epigenetic and DOHaD science.

## Conclusion

In this article, we have shown how scientists conceptualize epigenetic biomarkers as molecular vestiges of early life and how this produces novel epistemic objects and conceptualizations of health, risk, and intervention. In this way, scientists reinforce biomarkers as critical sites for interpreting how the social world shapes biology. This orientation is reflected in the opening epigraph, which describes how early life experiences can become biologically embedded, altering the body’s response to environmental experiences and exposures in ways that may shape health in later life. Our analysis shows how behavioral epigeneticists rely on this premise, the centrality of biomarkers in their work, and how these produce new environments of concern, even in the face of uncertainties. These findings illustrate persistent patterns and new directions in epigenetic research and reflect the need for postgenomic science scholars to attend to how this science relies on and extends previous approaches.

We have shown that the instability of knowing within epigenetic and DOHaD logics matters not only to knowledge practices at our sites but also as it generates novel epistemic objects aligned with broader trends in neoliberal capitalist societies around the persistent molecularization of health and illness, developments in biomarker discovery and validation, and the individualization of risk, intervention, and responsibility. In this context, the emergence of epigenetic research on PCEs specifically opens new sites of inquiry for social scholars of epigenetics and opportunities for self-reflection among epigenetic scientists themselves. These include questions about the growing landscape of epigenetic biomarkers, definitions of meaningful environments, and their application to various disorders, diseases, and health-related behaviors. Further, the emergence of PCEs as an epistemic object also begs further research into the responsibilities these may produce and how they will shape the diagnostics and interventions—pharmaceutical, familial, social, structural, and otherwise—that will undoubtedly be developed in their name. What new forms of health, risk, and responsibility may emerge from this focus is an open question that deserves attention in future STS scholarship.

## Data Availability

The data that support the findings of this study are available on request.
